# Human papilloma virus identification in ocular surface squamous neoplasia by *p*16 immunohistochemistry and DNA chip test: A strobe-compliant article

**DOI:** 10.1097/MD.0000000000013944

**Published:** 2019-01-11

**Authors:** Tina Shrestha, Won Choi, Ga Eon Kim, Jee Myung Yang, Kyung Chul Yoon

**Affiliations:** a Department of Ophthalmology and Research Institute of Medical Sciences; b Department of Pathology and Research Institute of Medical Sciences, Chonnam National University Medical School and Hospital, Gwangju; c Graduate School of Medical Science and Engineering, Korea Advanced Institute of Science and Technology, Daejeon, South Korea.; d Department of ophthalmology, Dhulikhel hospital, Kathmandu university school of medical science, Nepal.

**Keywords:** DNA chip test, human papillomavirus, immunohistochemistry, ocular surface squamous neoplasia

## Abstract

The aim of this study was to identify the association between human papilloma virus (HPV) infection and ocular surface squamous neoplasia (OSSN) using *p*16 immunohistochemistry (IHC) and deoxyribonucleic acid (DNA) chip test.

Thirty-eight patients who underwent surgical excision of OSSN were retrospectively studied using tissue samples. The IHC was performed to assess the expression of *p*16 and DNA chip test was used to detect 24 HPV serotypes.

Among the 38 OSSN samples, 32 cases (84.2%) were histopathologically categorized as pre-invasive type and 6 cases (15.8%) as invasive type. The IHC for *p*16 showed strong positivity in 12 cases (31.6%), whereas it was negative in 26 cases (68.4%). On the other hand, only one case (2.6%) of invasive OSSN was positive for the HPV16 serotype, as assessed by DNA chip test.

In OSSN, *p*16 expression was positive in approximately 1/3rd of the cases, whereas the majority of the 24 HPV serotypes were negative for *p*16. Our findings suggest that only a weak association exists between HPV infection and OSSN.

## 1 Introduction

Ocular surface squamous neoplasia (OSSN) is defined as a range of diseases from mild dysplasia to carcinoma in situ and invasive squamous cell carcinoma (SCC).^[1]^ The OSSN may be located in the cornea, conjunctiva, and limbus but is mostly found at the interpalpebral nasal limbus. The pathogenesis of OSSN is poorly understood and the role of various factors such as advanced age, male sex, vitamin A deficiency, and human immunodeficiency virus (HIV) and human papilloma virus (HPV) infection has not been definitely established yet.^[2]^ Conjunctival SCC represents the most severe form of OSSN, may associate with significant morbidity and also lead to mortality if left untreated.^[3]^ The global age-standardized incidence of OSSN is 0.18 annual cases per 100,000 males and 0.08 annual cases per 100,000 females.^[1]^

The HPVs mostly cause cervical, anal, and oropharyngeal cancers, as well as a relatively low number of cases of vaginal, vulvar, and penile cancers.^[4]^ The HPV consists of a small, non-enveloped, epitheliotropic strand of deoxyribonucleic acid (DNA). It can infect multiple types of epithelia, including stratified squamous cells as well as mucosal layers. The HPV contributes to cancer pathogenesis by the formation of a protein complex between the host p53 and the HPVE6 protein, resulting in blockage of the p53 suppressor action.^[5]^ The neutralization of the cellular retinoblastoma tumor suppressor (pRB) and the p53 tumor suppressor proteins by the HPV E6 and E7 oncogenes induces the expression of *p*16.^[6,7]^ Hence, *p*16 expression is considered a marker of high-risk HPV serotype infection.

The DNA chip test is a diagnostic tool. It is polymerase chain reaction (PCR)-based microarray technique that has an ability to simultaneously detect up to 24 HPV subtypes, including high-risk types. The sensitivity of HPV detection by DNA chip test in cervical specimens was 91.1%.^[8]^ Moreover, the sensitivity and specificity of the HPV DNA chip test in detecting HPV 16 and 18 have been proved to be as high as those of the Hybrid Capture 2 test.^[9]^ To the best of our knowledge, no other previous studies have evaluated 24 HPV serotypes in OSSN.

Previous studies focusing on the prevalence of HPV in OSSN showed conflicting results and a high degree of variability, with prevalence ranging from 0 to 100%.^[10]^ Moreover, only a limited number of HPV serotypes have been studied till date. Therefore, the purpose of our study was to evaluate the association of OSSN with HPV using immunohistochemistry (IHC) for *p*16 and the DNA chip test for the evaluation of 24 HPV serotypes.

## 2 Methods

Thirty-eight cases histologically diagnosed as OSSN from 2006 to 2016 were studied. Formalin-fixed, paraffin-embedded (FFPE) tissue blocks and glass slides were obtained from the Department of Pathology, Chonnam National University Hospital. A search of the Massachusetts Eye and Ear Infirmary/Massachusetts General Hospital pathology information system was performed. Histomorphologic characteristics were evaluated on hematoxylin and eosin–stained slides to verify the pathologic diagnosis. The FFPE tissues were cut into 5 μm-thick sections and processed for IHC and DNA chip test. This observational, retrospective study was approved by the Institutional Review Board of the Chonnam National University Hospital. No other treatment was done before surgical excision of OSSN.

### 2.1 Immunohistochemical evaluation of *p*16

The IHC staining for *p*16 was conducted in all 38 FFPE sections as per Manufacturer's instructions. Ventana detection kits (CINtec Histology, mtm Laboratories AG, Germany) were used with a Ventana Benchmark ULTRA auto-stainer (Roche Ventana Medical Systems Inc) to detect a mouse monoclonal antibody against *p*16 (E6H4 clone, CINtec Histology; Ventana Medical Systems, Tucson, AZ). A cervical SCC sample was used as a positive control for *p*16 expression. The *p*16 expression was considered positive in the presence of a continuous, diffuse cell staining in the basal and parabasal cell layers of the squamous epithelium and was considered negative in case of focal or no staining. A 70% staining within nucleus and cytoplasm was used as a threshold for *p*16 positivity. This percentage was selected because it best correlated with the HPV status in non-ophthalmic head and neck squamous tumors.^[11]^

### 2.2 HPV genotyping by DNA chip test

As per manufacturer's manual instructions HPV genotyping was performed, using a PCR-based DNA microarray system, by the DNA chip test (MyGene Company), comprising probes for 15 high-risk serotypes (HPV-16,18, 31,33, 35,39,45,51,52,53,56,58,59,66, and 68) and 9 low-risk serotypes (HPV-6,11,34,40,42, 43,44,54, and 70). Briefly, the procedure was carried out by isolating DNA from samples using a DNA isolation kit (MyGene Company), and amplified by PCR. The PCR products of 150 base pairs were labeled with a single dye, indocardocyanine-dUTP (MEN Life Science Products, Inc, Boston, MA), using consensus GPd5+/GP6d+ primers, and subjected to electrophoresis in a 2.5% agarose gel. The HPV–amplified material (10 μl) was denatured at 95°C for 5 min, mixed with hybridization solution (MyGene Company), and then applied to the DNA chip. Hybridization was performed for 90 min at 43°C followed by 5 min washing with 3X saline-sodium phosphate-ethylenediaminetetraacetic acid and drying at normal room temperature. The DNA chip scanner (ScanArray Lite; GSI Lumonics, Ottawa, ON) was used to visualize hybridized DNA signals. The HPV amplicons were hybridized with the corresponding type-specific oligonucleotide probes and visualized on HPV DNA chip slides as double positive spots when the HPV DNA was present in the PCR product. Negative controls (without DNA) showed no HPV positivity and did not produce any spot.

## 3 Results

**Figure 1:**
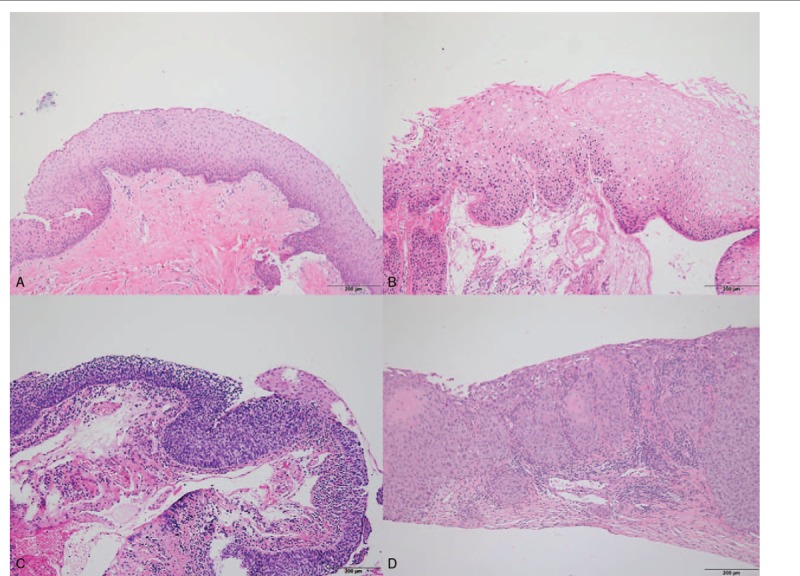
Histologic appearance of pre-invasive and invasive ocular surface squamous neoplasm. (**A**) Benign squamous hyperplasia. The epithelium is thickened, but shows normal maturation and no nuclear atypia. (**B**) Dysplasia. Atypical epithelial cells are seen but abnormal cells with nuclear atypia and abnormal maturation confined to the basal 3rd of the epithelium. (**C**) Squamous cell carcinoma in situ. The epithelium shows full-thickness atypia and polarity of the epithelium is lost. There is no invasion of the subepithelial tissue. (**D**) Invasive squamous cell carcinoma. Invasion by malignant epithelial cells present through the basement membrane into the subepithelial tissue.

A total of 38 FFPE samples diagnosed as OSSN were analyzed (Table 1). The mean age of the patients was 69.1 ± 12.0 years (range, 12–88 years), with 27 males (71.0%) and 11 females (29.0%). All cases presented with lesions of the conjunctiva. None of them were diagnosed as human immunodeficiency virus (HIV)-positive. The mean follow-up period was 2.63 ± 1.21 years. Histopathological examination classified 32 cases (84.2%) as pre-invasive lesions and 6 cases (15.8%) as invasive OSSN/squamous cell carcinomas. Pre-invasive lesions were further classified as mild (less than a 3rd thickness occupied by atypical cells) squamous dysplasia in 8 cases (21.1%), moderate (3 quarters occupied by atypical cells) squamous dysplasia in one case (2.6%), severe (nearly full thickness occupied by atypical cells) squamous dysplasia in 9 cases (23.7%), and squamous cell carcinoma in situ (loss of normal cells layer by atypical cells) in 14 cases (36.8%) f.^[12]^ Invasive OSSN/squamous cell carcinoma showed nests of neoplastic cells penetrating the epithelial basement membrane and spreading into the underlying stroma. Dysplasia of the conjunctival epithelium is determined by cellular pleomorphism, loss of cellular polarity, and acanthosis. With regard to the extent of the epithelial dysplasia, it can be devided to mild, moderate, or severe. Full thickness epithelial dysplasia without rupture of epithelial basement membrane is known for carcinoma in situ (Fig. 1).

**Table 1:**
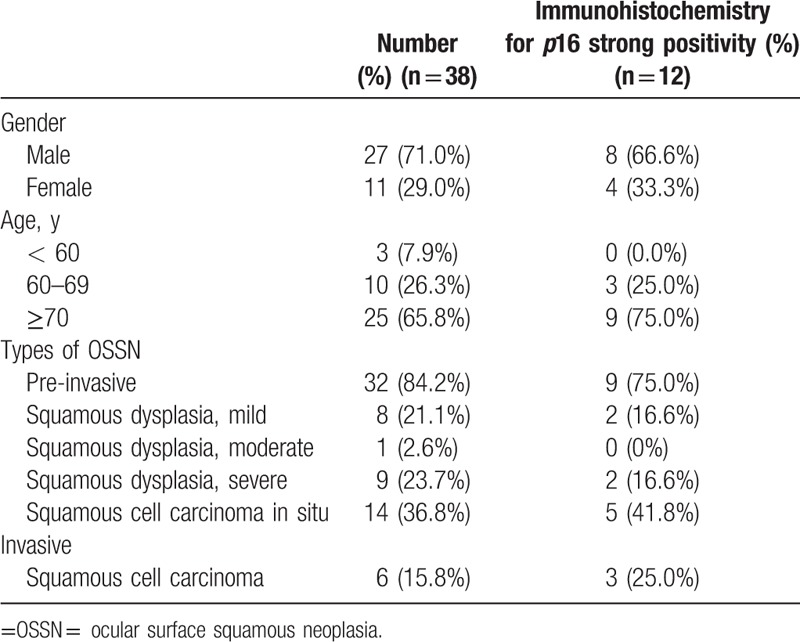
Clinicopathological and immunohistochemical characteristics of patients with ocular surface squamous neoplasia.

**Figure 2:**
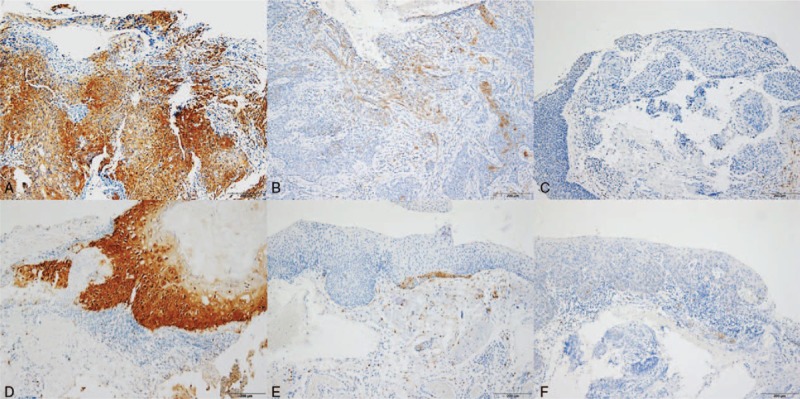
*p*16 immunohistochemistry of invasive (**A**, **B**, **C**) and pre-invasive (**D**, **E**, **F**) ocular surface squamous neoplasm. Representative photographs of positive (**A**, **D**), focally or weakly positive (**B**, **E**), and negative (**C**, **F**) *p*16 staining.

The 38 FFPE samples were assessed for HPV status based on immunohistochemical detection of *p*16 and DNA chip tests (Tables 2 and 3). The IHC for *p*16 was definitively positive in 12 cases (31.6%) and negative in 26 cases (68.4%). The latter samples comprised 20 cases (52.6%) of bona fide negative staining and 6 cases (15.8%) of weakly positive samples (Fig. 2). Representative photographs of hematoxylin and eosin and *p*16 IHC in the invasive and pre-invasive groups are shown in Figures 1 and 2, respectively. The HPV DNA chip test was performed twice on each of the 38 specimens, and we detected only 1 positive case (2.6%), represented by an HPV 16 serotype (Table 3).

**Table 2:**
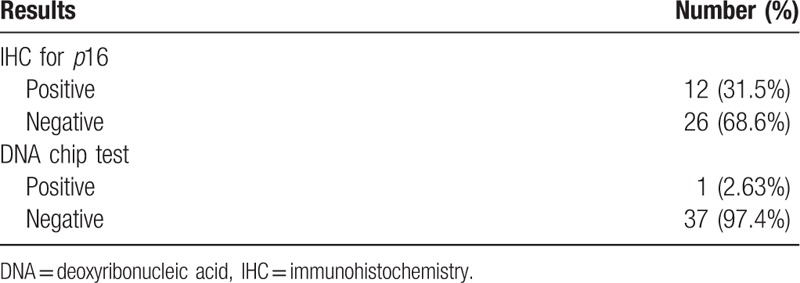
Results of human papillomavirus identification by immunonohistochemistry and DNA chip test_(n = 38).

**Table 3:**
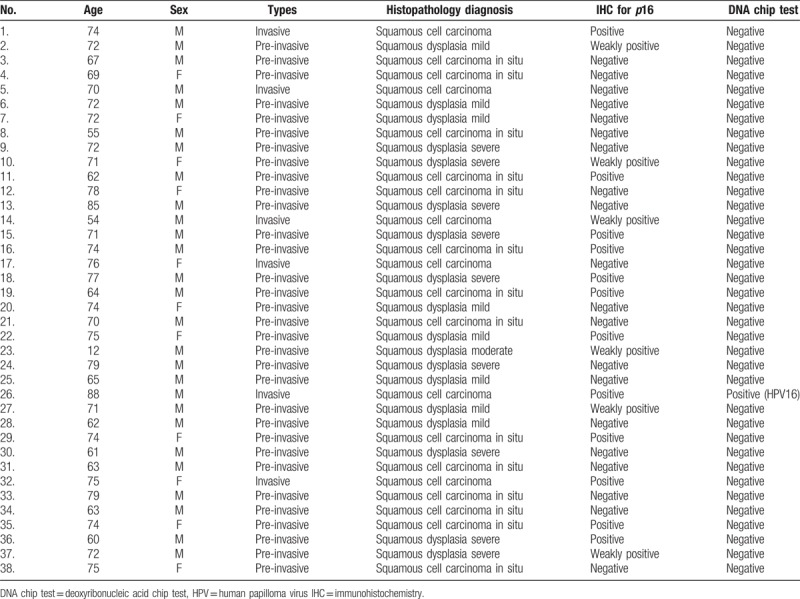
Summary of data: characteristics of individual patients and tumors, along with *p*16 and human papillomavirus evaluation.

## 4 Discussion

In this study, we retrospectively investigated the association between HPV and OSSN. The HPV prevalence in OSSN was found to be 31.6% and 2.6%, as assessed by *p*16 staining and DNA chip test, respectively. Only 1 case (2.6%) of invasive OSSN was HPV positive for the high-risk serotype 16. Of the 12 cases with strong *p*16 expression, 11 did not contain any HPV serotype, as determined by DNA chip test. Consistently, studies conducted in India and Germany showed no presence of high-risk HPV in OSSN.^[13,14]^ The HPV prevalence in OSSN is still controversial. According to previous studies, the average prevalence of HPV in OSSN is 33.8%, with values ranging from 0 to 100%.^[10]^ Most studies were based on small numbers of patients and employed a limited number of viral detection, e.g., PCR and in situ hybridization. Additionally, geographical differences, insufficient consensus on appropriate detection algorithms, and diverse genetic susceptibility may account for these discrepancies.

Cervical and nasopharyngeal cancer studies have established IHC detection of *p*16 as a suitable method for the assessment of HPV-positive neoplasia.^[15]^ However, many histopathologists, highly experienced in HPV diagnosis, warn against the pitfalls of *p*16 IHC in clinical practice.^[16,17]^ Concerning OSSN, the sensitivity of *p*16 expression as a method for HPV detection is questionable. A study conducted in Africa found strong *p*16 immunoreactivity in 67% of the examined conjunctival squamous cell carcinomas, whereas an Australian study on corneal and conjunctival squamous lesions only reported a 6.5% positivity.^[18,19]^ Analogously, Kobalka et al^[20]^ were not able to detect the HPV types 6, 11, 16, and 18 in any of the *p*16-positive cases evaluated. Our data showed that *p*16 expression is not a reliable indicator of the presence of HPV in OSSN, unlike cervical and nasopharyngeal squamous cell carcinoma, and suggested that HPV has a minor role in OSSN tumorigenesis.

The high-risk HPV serotypes 16 and 18 are currently considered as the most strongly associated with OSSN but other HPV serotypes (6, 11, 31, 33, 35, 45, 51, 52, and 66) have also been detected within these tumors.^[21]^ Previous studies have investigated a limited number of HPV serotypes, i.e., HPV 16 and HPV 18, with low-sensitivity methods. Therefore, the presence of HPV serotypes other than 16 and 18 may have been overlooked, resulting in false negatives. In order to more thoroughly address the involvement of HPV in OSSN pathogenesis, we employed the DNA chip test as it could detect 24 HPV serotypes and multiple infections at once by PCR-microarray-based method. The accuracy of HPV serotyping by this method has been certified by sequencing data in a previous study conducted on cervical lesions.^[8]^

The low prevalence of HPV infection in OSSN (2.6%), despite *p*16 positivity in 1/3rd of the cases, suggests that HPV infection may not be the cause but rather a risk contributor for OSSN. Other pathways, downstream cell cycle regulation might induce *p*16 expression independently of HPV infection. For instance, aberrant growth signals and dysregulated cell divisions can also induce the expression of *p*16. Beside HPV infection, excessive ultraviolet radiation exposure and coinfections with oncogenic viruses, including Epstein-Barr virus, cytomegalovirus, HIV, and Herpes simplex virus are considered as possible triggering factors for OSSN development.^[22–25]^ For example, HIV-related immunosuppression is considered as a risk factor of OSSN, as HPV infection is associated with an increased incidence of HIV acquisition.^[26]^

The present study has several limitations. First, it is a retrospective study with a low number of samples obtained from a single institute. Secondly, little information was retrieved about the clinical presentation and the follow-up of cases. Although the DNA chip test is one of the most comprehensive and convenient methods for HPV detection available to date, definitive tests based on the use of subtype-specific primers could help minimizing the risk of false-negative results.

In conclusion, although 1/3rd of the examined OSSN cases were positive for *p*16 expression, the great majority of the 24 HPV serotypes were not detected in any of the tumors, suggesting a weak association between HPV and OSSN. However, although HPV may not be necessary or sufficient to cause OSSN, it may still contribute to tumorigenesis. Further validation of these results is warranted in prospective studies to elucidate the role of *p*16 expression and the possible involvement of additional HPV serotypes in OSSN.

## Acknowledgments

The authors thank Dr. Punyaram Kharbuja, Department of Surgical Oncology, Bhaktapur Cancer Hospital, Nepal for his help in preparing the manuscript.

## Author contributions

**Conceptualization:** Ga Eon Kim.

**Data curation:** Tina Shrestha, Ga Eon Kim.

**Funding acquisition:** Kyung Chul Yoon.

**Investigation:** Tina Shrestha, Ga Eon Kim, Jee Myung Yang.

**Methodology:** Won Choi, Jee Myung Yang.

**Writing – original draft:** Tina Shrestha, Won Choi.

**Writing – review & editing:** Kyung Chul Yoon.
